# Infrared Transmission Characteristics of Phase Transitioning VO_2_ on Various Substrates

**DOI:** 10.3390/mi13050812

**Published:** 2022-05-23

**Authors:** Samee Azad, Durga Gajula, Nawraj Sapkota, Apparao Rao, Goutam Koley

**Affiliations:** 1 Holcombe Department of Electrical and Computer Engineering, Clemson University, Clemson, SC 29634, USA; gkoley@clemson.edu; 2 Georgia Institute of Technology, Atlanta, GA 30332, USA; dgajula@clemson.edu; 3 Department of Physics and Astronomy, Clemson University, Clemson, SC 29634, USA; nsapkot@clemson.edu (N.S.); arao@clemson.edu (A.R.)

**Keywords:** vanadium dioxide, transition resistance ratio, piezoelectric substrate, flexible substrate, transmitted optical power, infrared region, mechanical strain

## Abstract

Infrared transmission characteristics of VO_2_ thin films synthesized on multiple substrates, using a low-pressure direct oxidation technique, have been characterized. Material characterization of these films indicates high material quality, which resulted in large variation of electrical and optical properties at phase transition. A change in optical transmissivity greater than 80% was observed for these films utilizing infrared (IR) laser illumination at 1550 nm. Phase transition enabled by temperature change induced by a pulsed high-power laser beam resulted in modulated IR laser transmission with a low time constant in VO_2_ on transparent quartz and muscovite substrates. Investigation of the effect of mechanical strain on phase transition in VO_2_ grown on flexible muscovite substrate indicate shift in transition temperature to higher for tensile and lower for compressive strains.

## 1. Introduction

VO_2_ is a highly intriguing material that has attracted researchers over the last few decades. The easy-to-induce phase transition property of VO_2_ [1] has made the compound highly reliable for many applications, such as temperature and infrared (IR) sensing [2], smart windows [3], and for temperature based optical switches for waveguides at RF frequencies. During the phase transition, VO_2_ undergoes changes in electrical properties, optical properties, and structural properties. The reversible change of optical properties of VO_2_ is becoming a point of interest recently, because of the sharp and abrupt change of optical power transmitted through VO_2_ or reflected by VO_2_ during phase transition [4]. When a VO_2_ thin film is subject to being exposed to electromagnetic waves (UV, visible light, near-IR, IR, mid-IR), some of the optical power carried by the beam transmits through the VO_2_ film, a portion of it is reflected, and the rest of the power is absorbed by the VO_2_ film [5]. When the semiconductor to metal transition (SMT) initiates, the percentage of the transmitted optical power undergoes a sharp change to another level. The direction of the change depends on whether the VO_2_ is transitioning its state to semiconductor or to metal. It also depends on the wavelength of the light transmitted through it. At visible wavelengths, the transmission of light increases through VO_2_ when the film is transitioning from semiconductor to metal [6], and vice versa. After crossing a wavelength threshold, when the wavelength is near IR or IR, the transmission of light through VO_2_ starts to decrease when the film is transitioning from semiconductor to metal, and vice versa. Taha et al. [7] reports the percentage of the decrease of optical power as 42% for a wavelength at 1550 nm, and 60% for a wavelength at 2000 nm. Moreover, VO_2_ on quartz shows similar characteristics which have been observed by other researchers (i.e., 40.9% at 1550 nm [8]). They also report 48.8% of change when the laser is of a wavelength of 1800 nm. At 1550 nm, the transmission loss through the optical fiber (glass) is minimal, which makes this wavelength very attractive for optical communication, as widely reported [9]. The results from the literature provides an estimate of what type of results we should expect if we use the telecommunication wavelength 1550 nm laser beam to transmit through the VO_2_ thin film. In order to observe the phenomenon, first we synthesize VO_2_ thin films on five types of substrates (sapphire, SiO_2_/Si, AT-cut quartz, GaN/AlGaN/GaN/Si, and muscovite) using a low-pressure direct oxidation method [10].

Explaining VO_2_ behavior on very dissimilar substrates such as these is not straightforward; however, we did manage to optimize the film quality, especially focusing on the resistance transition ratio, optical transmittance change, and transition temperature. We utilized sapphire and SiO_2_/Si substrates for validation and benchmarking the quality of the VO_2_ thin films by comparing them with the existing literature (where these substrates are most commonly used for VO_2_ synthesis). On the other hand, AT-cut quartz and GaN/AlGaN/GaN/Si substrates were selected because of their piezoelectric properties, which can enable the VO_2_ films to change phase based on strain changes, as well as offer potential for integration with versatile and significant device applications utilizing these substrates. Finally, muscovite was selected for its flexibility and corresponding utility as a test platform for enabling phase change in VO_2_ through strain.

In this work we have utilized the 1550 nm telecom wavelength laser to determine the variation in optical transmissivity of VO_2_, induced by phase transition. The flexibility of the muscovite substrates enabled us to apply both a compressive and tensile strain on it [11] and study their effect on the transition temperature [12]. The work embodies a technological demonstration of the growth of high-quality films on piezoelectric substrates through a rational optimization process. Furthermore, technological significance of growth on AT-cut quartz and GaN/AlGaN/GaN/Si is to study the strain induced phase transition, and the potential for future integration with versatile devices on these substrates, including AlGaN/GaN high electron mobility transistors (HEMTs), [13] and surface acoustic wave (SAW) devices on both quartz and III-Nitrides [14,15].

## 2. Experimental Details

### 2.1. VO_2_ Synthesis

**Figure 1:**
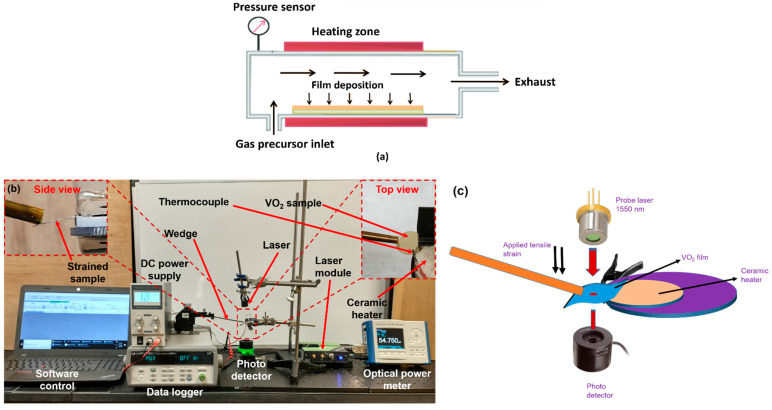
(**a**) Schematic for the synthesis process of VO_2_ thin film by direct oxidation (**b**) Experimental setup for electrical and optical characterizations of the VO_2_ thin films. The laser is shone from the top, while a photodetector at the bottom (along with a power meter) measures the transmitted laser power as phase transition occurs. Inset shows a magnified image of the white annular ceramic heater with the sample on edge pressed and bent with a wedge. (**c**) A basic schematic of the experimental setup, including the VO_2_ film sample, ceramic heater, laser, photodetector and strain applying wedge.

The synthesis of the VO_2_ films were achieved by using a homemade low-pressure furnace (Figure 1a) through the controlled oxidation of vanadium thin films of desired thicknesses deposited on the five types of substrates (c-plane sapphire, SiO_2_/Si (100), AT-cut quartz, or GaN/AlGaN/GaN [c-plane or (0001) plane])/Si (111) [10]. Prior to growth, the substrates were coated with vanadium thin films, using high purity (99.7%, from Kurt J. Lesker) vanadium pellets with a deposition rate of 1.5 A°/s in an electron beam evaporation system. Chamber pressure is expected to reach 4.53 Pa under pump down, N_2_ gas (purity 99.999% from Airgas) flow was started at a constant rate of 400 sccm until the chamber pressure stabilized at ~2666 Pa. The furnace temperature was then increased to 475 °C, and O_2_ flow was initiated at the rate of 100 sccm. After optimization, synthesis parameters for VO_2_ thin films (starting with 70 nm vanadium deposition on the various substrates are summarized in Table 1. The optimization was decided based on the VO_2_ thin films attaining specific benchmarks in terms of quality, which includes the resistance transition ratio, optical transmittance change, and transition temperature. The typical target transition resistance ratio is > 400, it has an optical transmittance of > 80%, and transition temperature in the range of 60–75 °C. We would like to mention here that although the nature of the substrate can significantly affect the film quality, our synthesis method, with proper optimization, was able to produce VO_2_ thin films of high quality as manifested by the transition resistance ratio, transmission percentage change, and transition temperature, which clearly underlines the utility and potential of this technique. The pressure and duration of oxidation were found to be the most significant parameters, optimized over several growth iterations, to ensure high quality of the VO_2_ films for all the substrates mentioned above, carefully avoiding under-oxidation and over-oxidation.

### 2.2. Characterization Techniques

Optical microscopic images of the VO_2_ thin films were captured using microscope Olympus BX41M-LED at 50× magnification. For structural characterization and analyzing the surface characteristics, tapping mode atomic force microscopy (AFM), Veeco Dimension 3100 was used and the data were processed using the AFM software application [10].

VO_2_ samples synthesized on the five substrates were subjected to X-ray diffraction (XRD) measurements (Rigaku Ultima IV system), using Cu-Kα radiation (wavelength 1.5406 Å) where the diffracted beam was recorded from 5° to 90° with a step size of 0.02°, for determining the purity of the films [10].

For identification of the phase and the Raman modes, we collected Raman spectra using a Renishaw InVia micro-Raman spectrometer (10× objective) with a 532 nm diode laser (Crystalaser), at 50% of the maximum laser power. We vary the Raman shift from 100 to 1000 cm^−1^ for three different spots on each of the sample [16,17].

During the semiconductor to metal transition (SMT), the VO_2_ thin films undergo changes in surface resistance (sample temperature is varied from 20 to 120 °C), which was measured using the setup shown in Figure 1b,c. The surface of the VO_2_ thin film has two connected probes for the measurement of resistance, and it is connected to a Datalogger (Keysight 34972A LXI Data Acquisition Unit). An annular ceramic heater was used to apply heat to the sample within the preferred range measurement temperature. The VO_2_ sample has a thermocouple connected to it, to ensure the simultaneous accurate measurements of a sample temperature and thin film resistance [10].

Transmitted optical power variation with temperature is investigated, as is the electrical characterization to determine changes in optical properties associated with SMT. Transmitted optical power through VO_2_ thin films undergoes a sharp transition during SMT, which is significantly higher for infrared light compared with visible light. Similarly to the resistance change, the transmitted optical power was also measured by spanning across the SMT for the VO_2_ thin films as a function of temperature and using the similar characterization setup as shown in Figure 1. The VO_2_ sample was attached to an annular ceramic heater and placed in-between an IR laser and a photodetector (Newport 918D-IR-003R, range 780 to 1800 nm) in a vertical setup arrangement for measuring the transmitted light power using a Newport 1918-R power meter [10].

## 3. Results and Discussions

### 3.1. Material Characterization

**Figure 2:**
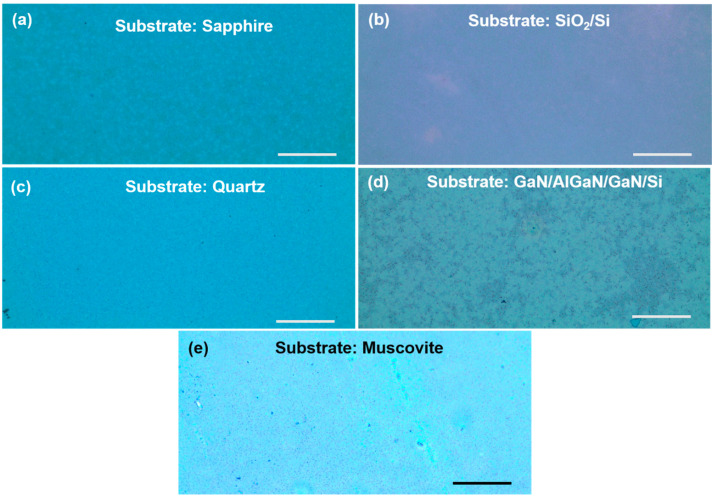
Optical images (50× magnification) of VO_2_ thin films (5 mm × 3 mm) synthesized from 70 nm vanadium deposited on a (**a**) c-plane sapphire, (**b**) SiO_2_/Si, (**c**) AT-cut quartz, (**d**) GaN/AlGaN/GaN/Si, and (**e**) Muscovite substrates. The scale bar is 500 µm for all substrates.

**Figure 3:**
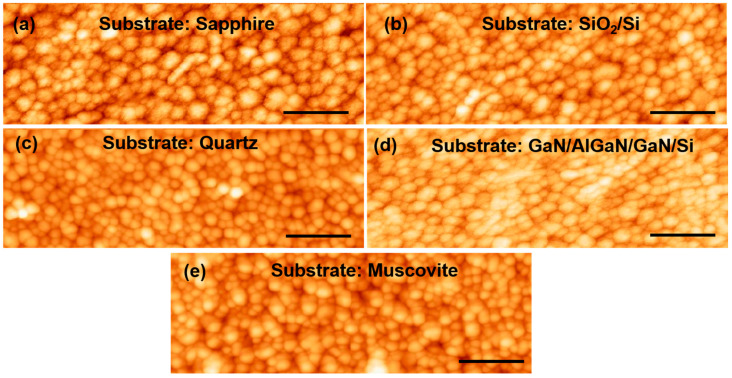
Surface morphology images (5 µm × 2.5 µm) of thin films synthesized from 70 nm vanadium deposited on (**a**) c-plane sapphire (z-scale bar 58.1 nm), (**b**) SiO_2_/Si (z-scale bar 50.4 nm), (**c**) AT-cut quartz (z-scale bar 75.4 nm), (**d**) GaN/AlGaN/GaN/Si (z-scale bar 68.1 nm), and (**e**) muscovite (z-scale bar 88.5 nm) substrates. The scale bar: 200 nm for all substrates.

Figure 2 shows the optical microscopic images of the VO_2_ samples grown on the five substrates (i.e., sapphire, SiO_2_/Si, quartz, GaN/AlGaN/GaN/Si and muscovite). The bluish-green and purple color of VO_2_, in comparison with what is mentioned in the literature [18], is also clearly noticeable in all four images shown in Figure 2. An atomic force microscope (AFM), Veeco Dimension 3100, was utilized to capture surface morphology images (5 × 2.5 μm) of the VO_2_ samples synthesized on the five different substrates and are shown in Figure 3. From Figure 3, we find the films to be mostly uniform with some granularity, as expected for the polycrystalline thin film layers. In Table 2, the rms surface roughness values for VO_2_ samples grown on sapphire, SiO_2_/Si, quartz, GaN/AlGaN/GaN/Si, and muscovite are found to be 8.19, 7.37, 10.3, 9.75 and 12.2 nm, respectively. The consistent morphological quality of the VO_2_ thin films are indicated by the uniformity of the roughness numbers. Polycrystalline film of monoclinic grain structure can exhibit surface roughness comparable to what our samples have, as reported in the literature [19]. In addition, as reported by Lindstrom et al. there is a proportionate relationship between surface roughness and the oxidation time [20].

**Figure 4:**
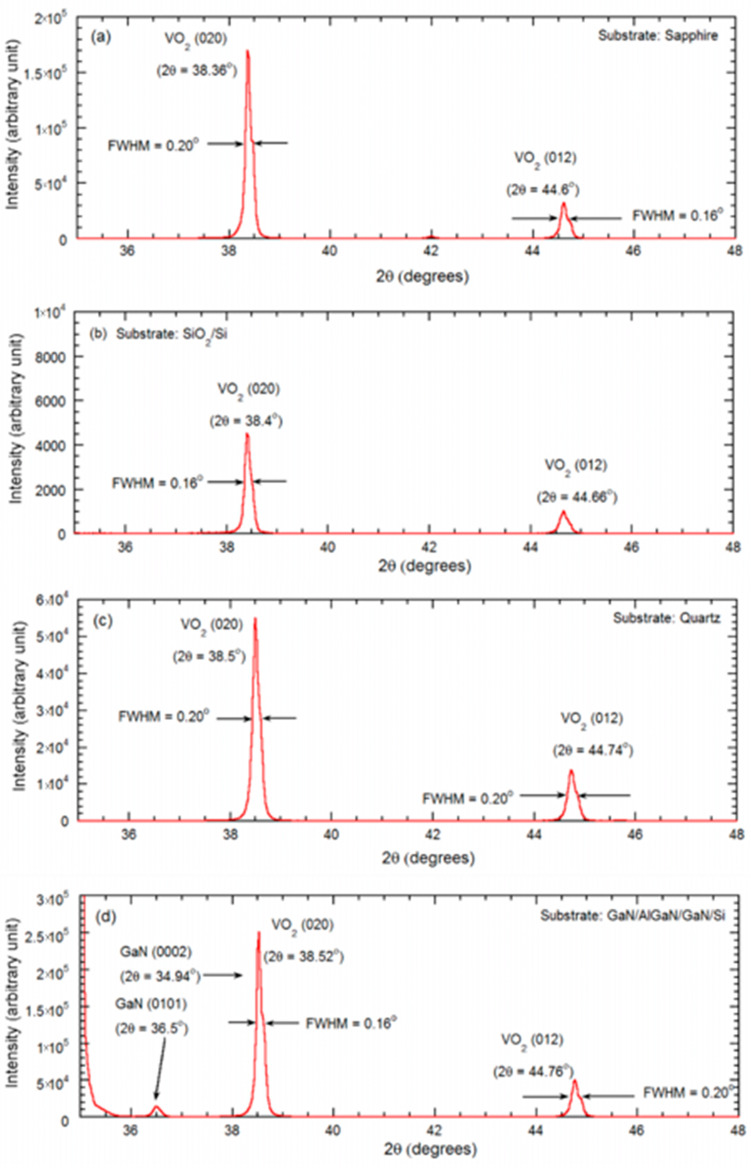

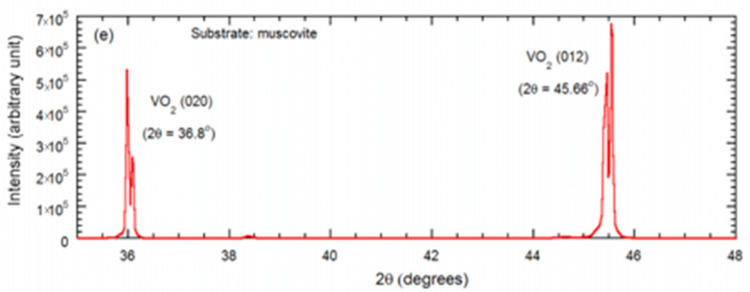
X-ray diffraction peaks are presented for the VO_2_ thin films synthesized on (**a**) c-plane sapphire, (**b**) SiO_2_/Si, (**c**) AT-cut quartz, (**d**) GaN/AlGaN/GaN/Si, and (**e**) muscovite substrates. The VO_2_ (020) and VO_2_ (012) peaks, along with their respective full width at half maxima (FWHM), are pointed out with arrows.

Analyzing the XRD scans and the diffraction angles, in Figure 4 we find the characteristic peaks for VO_2_ films synthesized on c-plane sapphire that are found at diffraction angles 38.36° for VO_2_ (020) and 44.6° for (012). For substrates such as SiO_2_/Si, prominent VO_2_ peaks are found at 38.42° for (020) plane and 44.66° for (012) plane. For VO_2_ (020) synthesized on quartz, high intensity peaks are observed at 38.5°, and 44.74°, for (012) plane. For substrates such as GaN/AlGaN/GaN/Si (111), VO_2_ intense diffraction peaks are observed at 38.52° for (020) and 44.76° for (012) plane, which is comparable to the existing literature (JCPDS card no. 44-0252) on different substrates [21,22,23].

A summary of the diffraction angle at the 2θ peak position and the full width at half maximum (FWHM) values for the prominent peaks are displayed in Table 2, where the high directionality of the polycrystalline domains in the VO_2_ films are indicated by the tight range of the FWHM (0.06°–0.20°).

**Figure 5:**
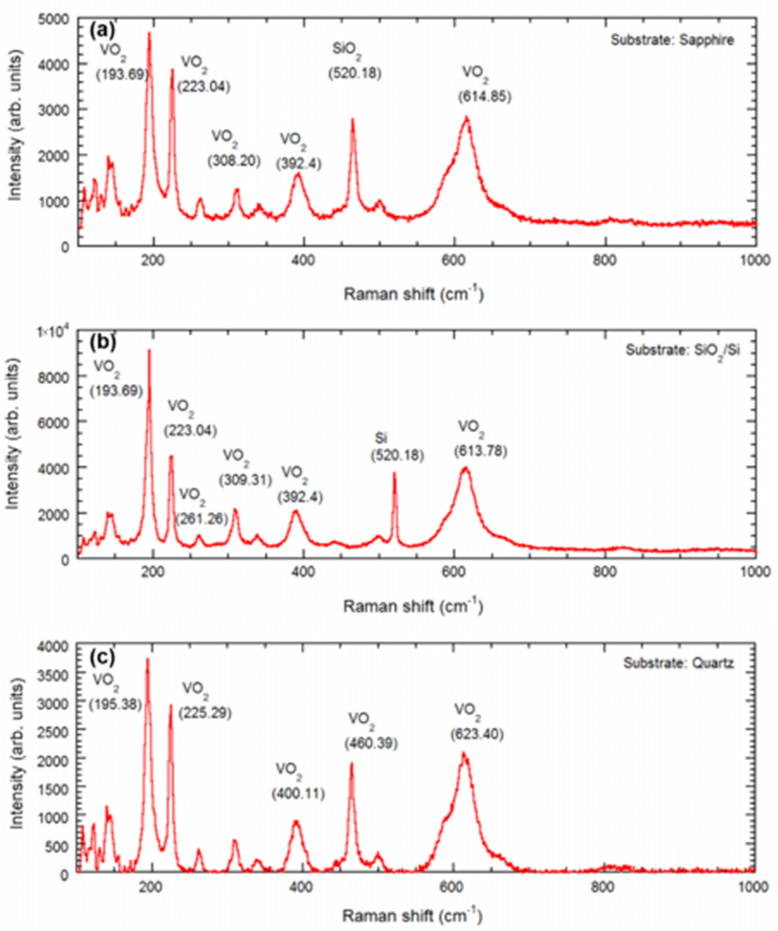

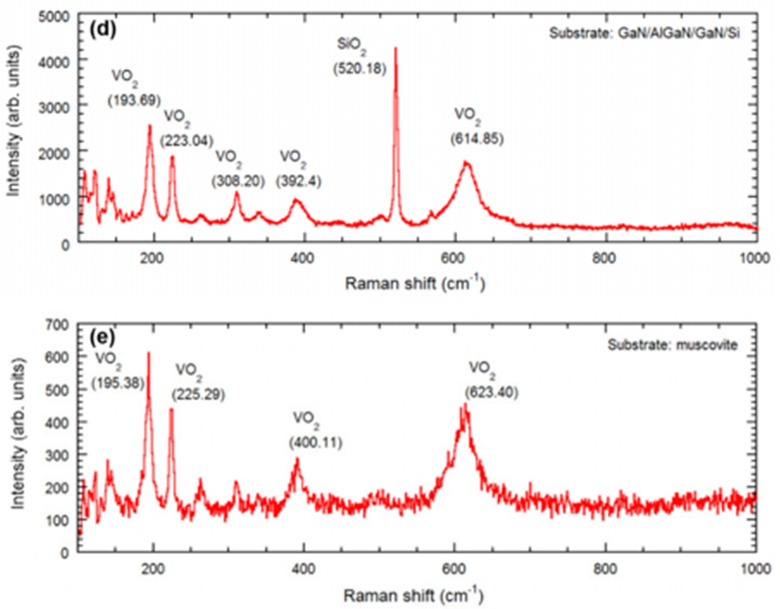
Raman peaks are presented for the VO_2_ thin films synthesized on (**a**) c-plane sapphire, (**b**) SiO_2_/Si, (**c**) AT-cut quartz, (**d**) GaN/AlGaN/GaN/Si, and (**e**) muscovite substrates. The VO_2_ (at 193 cm^−1^, at 223 cm^−1^, and 614 cm^−1^) common peaks.

Raman spectroscopy performed on the VO_2_ samples are shown in Figure 5. The Raman spectra on all the substrates displayed intensity peaks at Raman shifts ~195, 223, 395, and 614 cm^−1^, which correspond to VO_2_, and indicate the dominating presence of VO_2_ in the thin films synthesized on these substrates [16,17]. For the SiO_2_/Si and GaN/AlGaN/GaN/Si substrates, we find an additional intense peak at 520.18 cm^−1^, due to the Si substrate.

### 3.2. Electrical Characterization

**Figure 6:**
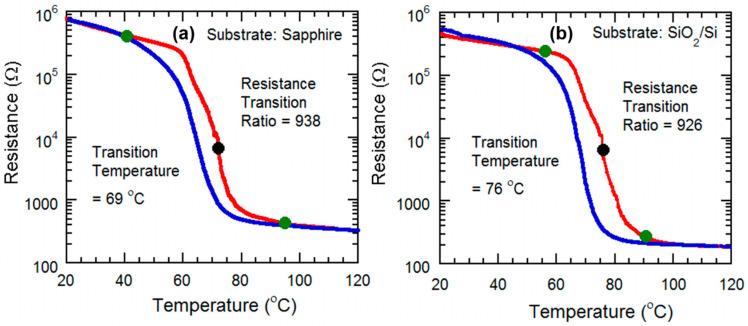

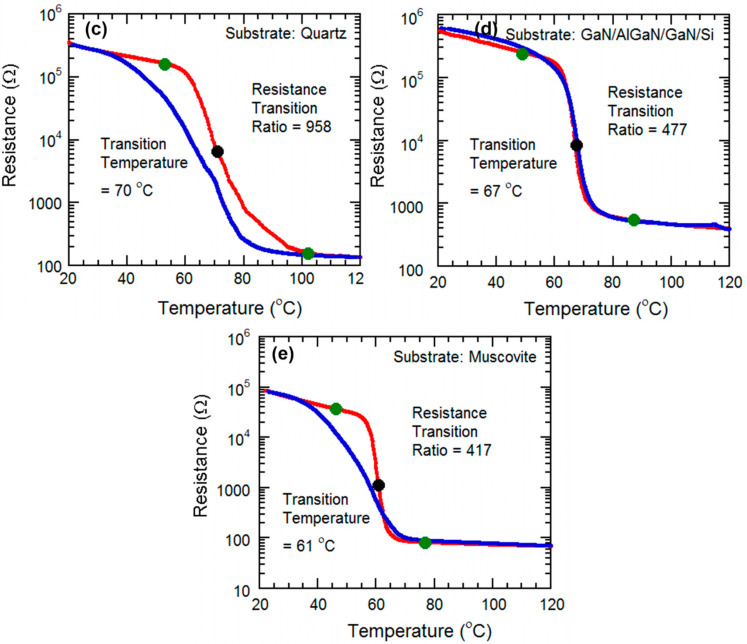
Semi-log plots of resistance variation as a function of temperature for the VO_2_ thin films grown on various substrates (**a**) c-plane sapphire, (**b**) SiO_2_/Si, (**c**) AT-cut quartz, (**d**) GaN/AlGaN/GaN/Si, and (**e**) muscovite substrates. as they undergo semiconductor-metal transition (SMT). The transition resistance ratios, along with the beginning (green dots), mid (black dots, corresponding to maximum slope points in the curves), and end transition temperatures (green dots) are shown for all the samples. The red line represents the forward phase transition curve, whereas the blue line indicates the reverse phase transition curve.

The experimental setup in Figure 1 has been utilized to observe the electrical resistance variation. A two-point probe measurement setup has been implemented to observe the effect of substrate heating on the electrical resistance of VO_2_ [10]. A ceramic heater is used to vary the temperature of the VO_2_ film, assisted by a thermocouple for recording the temperature, and tungsten probes connected to a data acquisition unit for measuring the surface resistance. The results are plotted in Figure 6, and summarized in Table 2, and are among the best reported in the literature, confirming the high quality of our VO_2_ thin film [23,24,25,26,27,28,29,30,31,32].

### 3.3. Optical Characterization

(i) Substrate heating by ceramic heater

Variation in transmitted optical power vs. temperature is often observed to identify and measure optical property changes due to SMT at telecom wavelength 1550 nm. During SMT, there occurs a sharp transition in transmitted optical power through VO_2_ thin films. Transmitted optical power was measured during the SMT for the VO_2_ thin films as a function of temperature, using the same characterization setup as shown in Figure 1.

**Figure 7:**
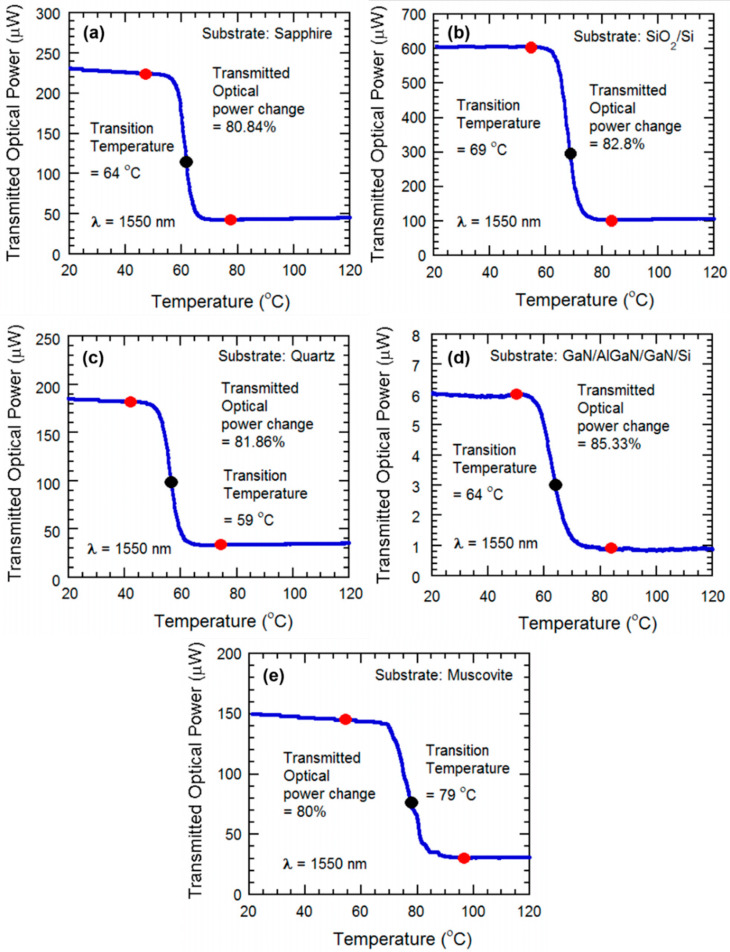
Transmitted optical power for IR laser wavelengths (1550 nm) plotted against temperature. Thin films grown on c-plane sapphire (**a**), SiO_2_/Si (**b**), AT-cut quartz (**c**), GaN/AlGaN/GaN/Si (**d**) and muscovite (**e**) as they undergo metal-insulator transition (SMT). The transmitted optical power change, along with the beginning (red dots), mid (black dots, corresponding to maximum slope points in the curves), and end transition temperatures (red dots) are shown for all the samples.

In Figure 7a–e we observed the experimental results on the transmitted optical power variation through the VO_2_ films grown on c-plane sapphire, SiO_2_/Si, AT-cut quartz, GaN/AlGaN/GaN/Si, and muscovite for the IR laser’s wavelength at 1550 nm. Transmitted laser power through the VO_2_ thin film undergoes a sharp change, since semiconducting VO_2_ allows IR light to transmit through it before SMT, whereas “metallic” VO_2_ film is reflective of the IR beam [33], and causes a sharp drop in the transmitted power. A reduction in transmitted laser power by approximately 80% for 1550 nm, was observed for the VO_2_ film on all the substrates. A summary of the optical properties of the films are presented in Table 2. We note that the transition in transmitted optical power is the highest reported so far at wavelength 1550 nm. The results compared to the existing literature are shown in Table A1, Appendix A.

We observe that optical transitions are sharper in Figure 7, and compared with the transition temperatures of electrical transitions (Figure 6), here, the transition temperatures are significantly lower. The reason is likely to be the fact that resistance of almost the entire VO_2_ film influences the electrical transition plots, and the thermal energy from the heater changes the temperature in a slower process. During optical transition, only the small area of the VO_2_ film with a focused laser beam influences the transmitted optical power. Since the transition of that small area is affected both by the thermal energy provided by the heater and the energy absorbed from the laser power focused on it, the transition happens much quicker, which is manifested as a much steeper transition slope and significantly reduced transition temperature.

(ii) Substrate heating assisted by high powered laser

Instead of realizing SMT using heating, an electric field, or strain, here, we have used a high-powered laser (124 mW, 635 nm) to induce the semiconductor to metal transition. The IR beam of 1550 nm probe laser was transmitted through the same high-power laser illuminated spot of the VO_2_ sample and detected using a photodetector underneath. The details of the characterization setup and the results are discussed later.

## 4. Synthesis and Characterization on a Flexible Substrate (Muscovite)

### 4.1. Synthesis

VO_2_ thin film was synthesized on 70 nm vanadium coated muscovite disks, utilizing the direct oxidation-based technique [10]. The synthesis parameters were optimized and described in Table 1. The surface and structural characterization were performed on VO_2_/muscovite samples by AFM, XRD, and Raman spectroscopy (Figure 2e, Figure 3e, Figure 4e and Figure 5e). All the characterization results indicate the presence of uniform and high quality VO_2_ in the sample, which predicts the high reliability of the characterization results after applying strain on the VO_2_/muscovite thin films [11].

### 4.2. Electrical Characterization

(i) Substrate heating without mechanical strain applied

The procedure and experimental setup are similar to the ones we used for the four other substrates beforehand. Silver conductive paste is used to form two stable terminals on the VO_2_ surface. The terminals are connected to the data acquisition unit which is used for measurements of resistance and temperature. The VO_2_ was placed on top of a ceramic heater, which is used for varying the temperature of the sample by heating or cooling it.

**Figure 8:**
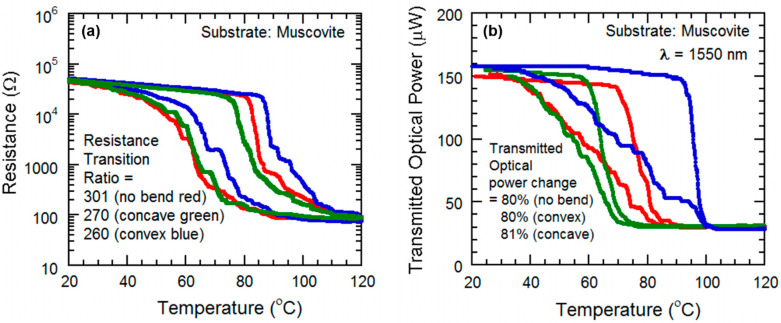
(**a**) Semi-log plots of resistance variation and (**b**) transmitted IR power as functions of temperature for the VO_2_ thin films grown on the muscovite substrate as they undergo a semiconductor–metal transition (SMT) during no mechanical strain (red), tensile strain (blue), and compressive strain (green).

The resistance of the VO_2_ thin film was measured with respect to the change of temperature from 20 °C to 140 °C, with an attached thermocouple to record the temperature. With an increase in heat, the VO_2_ starts changing its phase and conductivity, from semiconductor to metallic (see Figure 6e and Figure 8a). The transition ratio is observed to be 301 in Figure 8a, which is slightly lower than that observed in Figure 6e.

(ii) Substrate heating with tensile strain applied to the sample

For this we used a setup where, at the edge of the ceramic heater, the VO_2_ sample is attached to a clamp. The other portion of the sample is kept freely suspended. The suspended part of the VO_2_ thin film is bent in a convex way, by pressing down the sample with a screw-and-wedge as before. The strain was enough to ensure the convex bending of the VO_2_ is visible. The heater was used to apply heat on the convexly bent VO_2_ thin film, and the temperature was varied from 20 °C to 140 °C. As with the previous setup, the resistance is measured by the data acquisition unit. As the VO_2_ undergoes the SMT transition, the resistance decreases sharply. The effect of convex bending is observed after plotting the transmitted optical power data, we see that the ratio of change has decreased slightly to ~270 (Figure 8a). In addition, the transition region has shifted to the right, indicating that under tensile strain, the SMT for VO_2_ occurs at a higher transition temperature compared with the unstrained case.

(iii) Substrate heating with compressive strain applied to the sample

This characterization setup is similar to the one for the tensile strain study, but this time the sample is pressed upwards in the free edge, to ensure the concave bending of the sample. Again, the resistance varied with change in temperature, experiencing the steepest change at the SMT transition region of the VO_2_. After plotting the data, it is observed that ratio of change is ~260, but this time the transition region has shifted to the left, which means, while the VO_2_ is bent concavely, the SMT occurs at a lower transition temperature (Figure 8a).

### 4.3. Optical Characterization

(i) Substrate heating without mechanical strain applied

The procedure and experimental setup is similar to that used for the five other substrates beforehand. The photodetector and the IR laser were the same as before as well. The 1550 nm IR beam was transmitted through the VO_2_ sample placed in between the laser and the photodetector.

The transmitted IR power through the VO_2_ thin film is varied and measured with respect to the change in temperature from 20 °C to 140 °C. The heating induces semiconductor to metal transition in the VO_2_ thin film, which initiates changing its phase and conductivity, from semiconductor to metallic. At the transition region, the photodetector detects a sharp decrease of transmitted power, decreasing by ~85%, and return to initial level after being cooled down. The results compared to the existing literature are shown in Table A1, Appendix A.

(ii) Substrate heating with tensile strain applied to the sample

For this we used a setup similar to that used for optical characterization during tensile strain. At the edge of the ceramic heater, the VO_2_ sample is attached to a clamp. The suspended part of the VO_2_ thin film is bent in a convex way, by pressing down the sample with a screw-and-wedge. The tensile strain was enough to ensure that the convex bending of the VO_2_ was visible from Figure 1. The heater was used to apply heat on the convexly bent VO_2_ thin film. As with the previous setup, the IR beam is focused on the VO_2_ surface, and a portion of the IR laser power is transmitted though the sample and measured by the photodetector. By applying heat, the temperature is varied from 20 °C to 140 °C. The VO_2_ undergoes the SMT transition, causing the sharp decrease of transmitted optical power in the transition region. The effect of convex bending is observed after plotting the transmitted optical power data, and we see that the percentage of change has decreased slightly, but is still around ~80%. Moreover, we notice the transition plot has shifted to the right, indicating the increase in transition temperature due to tensile strain (Figure 8b).

(iii) Substrate heating with compressive strain applied to the sample

The setup is similar to the one used for tensile strain, but this time, the sample is pressed upwards in the free edge, to ensure the concave bending of the sample by compressive strain. Again, the IR power is transmitted through the thin film, and the power is varied due to the variation of the temperature, experiencing the highest percentage of variation at transition region of the VO_2_. After plotting the data, it is observed that percentage of change is still close to ~80%, but this time, the transition region has shifted to the left, indicating the decrease in transition temperature due to compressive strain. (Figure 8b)

(iv) Substrate heating assisted by high powered laser

**Figure 9:**
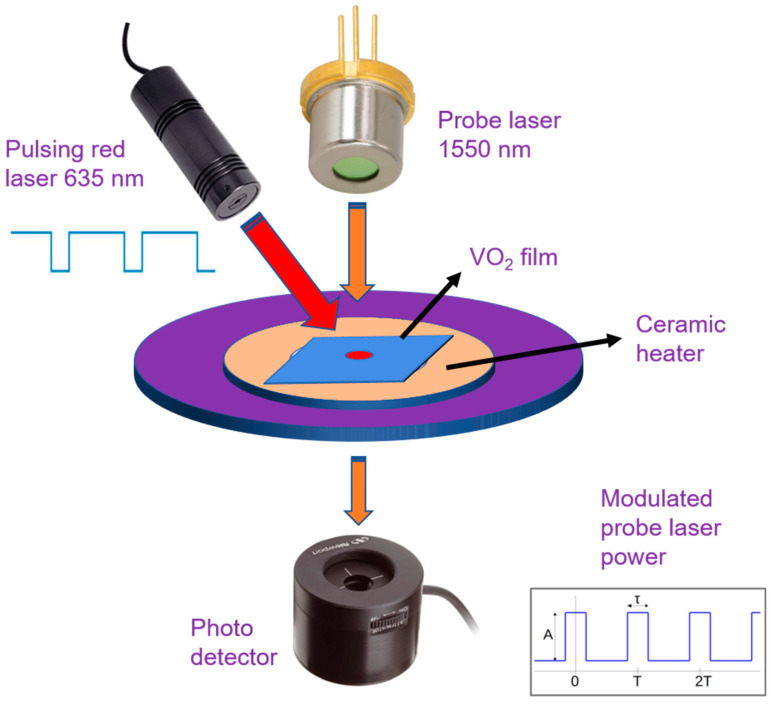
Schematic for inducing SMT in VO_2_ with a high-powered pulsed laser (124 mW, 635 nm) for modulating the probe laser power (1550 nm) transmitted through the VO_2_ thin film.

**Figure 10:**
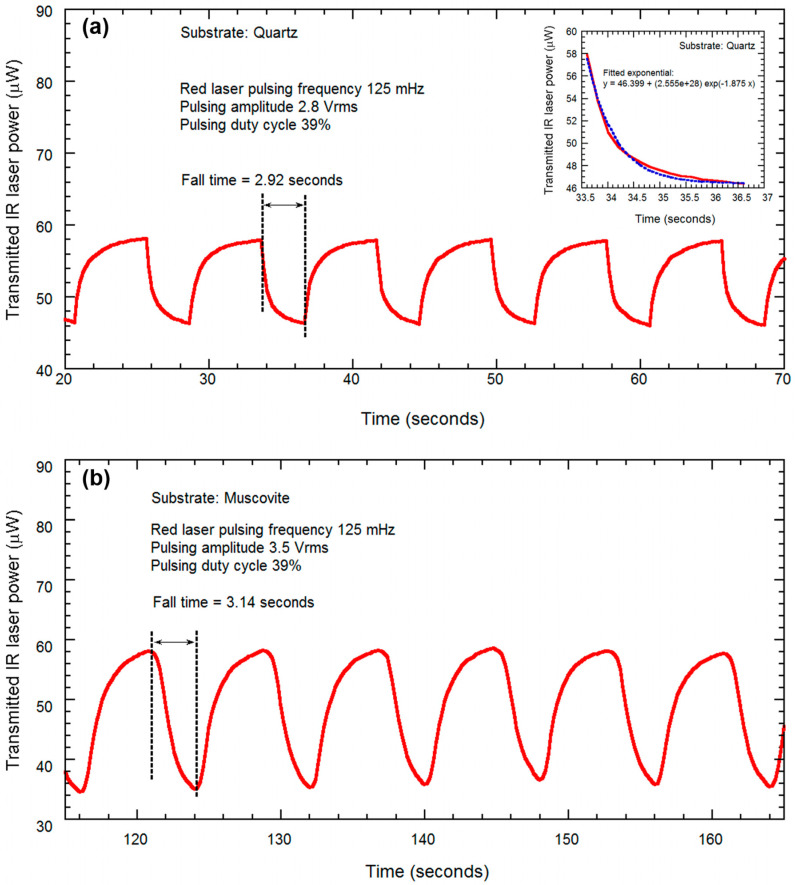
Modulation of transmitted optical power for IR probe laser wavelengths (1550 nm), plotted with respect to time, for the VO_2_ thin films grown on (**a**) quartz, falling time 2.92 s and (**b**) muscovite, falling time 3.14 s, undergo semiconductor–metal transition (SMT) due to pulsing of red laser (635 nm, 124 mW, 0.125 Hz and 39% duty cycle).

Finally, we utilized a high-power laser (124 mW, 635 nm) pulsed at 0.125 Hz (39% duty cycle) frequency to induce SMT in the VO_2_/muscovite sample, and plotted the response of the transmitted IR laser power, with the goal of studying the modulation of a probe laser power at the observed telecom wavelength of 1550 nm. A schematic diagram for the experiment is shown in Figure 9. We also performed a similar study on VO_2_/quartz film, in order to compare the characteristics of VO_2_/muscovite film. We used the ceramic heater to maintain a constant temperature closer to the transition temperature (50 °C for quartz and 45 °C for muscovite) to make it easier for the high-power laser to induce SMT. At each cycle of the red laser being pulsed, we observe that the IR power is pulsing from a high to low level of transmitted power. The higher level of power is transmitted when the VO_2_ is in semiconductor phase, whereas the reverse was observed in the metallic phase. The experimental results are shown for VO_2_ grown on quartz and muscovite substrates in Figure 10a,b, respectively. We find that although the change in transmitted IR power due to high power pulsing was ~25% for the VO_2_ on quartz substrate, it was ~40% for VO_2_ on muscovite. The fall time constants were found to be ~2.92 s and 3.14 s for the VO_2_ on quartz and muscovite substrates, respectively. Inset of Figure 10a shows determination of the fall time constant by least square fit of an exponential curve to the fall transient of the transmitted laser power. We would like to mention here that the need for an external heater can be eliminated using a higher-powered laser which can also reduce the switching time constant.

## 5. Conclusions

In conclusion, we have reported on the phase transition induced change in electrical and optical transmission (at 1550 nm) characteristics of VO_2_ films grown on multiple substrates utilizing a ceramic heater to change the sample temperature. The structural, electrical and optical characterization of the VO_2_ films underline their high quality and performance characteristics that are among the best results reported on films synthesized by other techniques on common substrates. The VO_2_ films synthesized on the piezoelectric GaN/AlGaN/GaN/Si and AT-cut quartz substrates exhibited excellent crystalline, morphological, and electrical properties, as well as a high resistance transition ratio and very high transmitted optical power change. In addition to the thermally induced SMT, we have demonstrated periodic VO_2_ phase transition induced by a high-powered red laser, underlining the possibility of localized and non-contact phase transition in these films. Furthermore, films were synthesized on a flexible muscovite substrate with excellent quality and performance metrics. Optical characterization of VO_2_ grown on muscovite shows a high percentage of decrease of transmitted IR laser power by ~80%, which, to our knowledge, is the best reported transmittance change so far at 1550 nm. Taking advantage of the flexibility of muscovite substrate, bidirectional mechanical strains, both compressive and tensile, were applied to the sample, resulting in the shift of phase transition plots to lower and higher transition temperatures, respectively.

## Figures and Tables

**Table 1:** Summary of the optimized material synthesis parameters for VO_2_ samples grown on five different substrates: sapphire, SiO_2_/Si, AT-cut quartz, GaN/AlGaN/GaN/Si and muscovite.

Substrate	Optimized VO_2_ Growth Parameters			
	Temperature (°C)	Pressure (Pa)	Oxidation Time (min)	Vanadium Thickness (nm)
c-plane sapphire	475 °C	5.2	50	70
SiO_2_/Si	475 °C	6	40	70
AT-cut quartz	475 °C	7.5	70	70
GaN/AlGaN/GaN/Si	475 °C	4.5	60	70
Muscovite	475 °C	4.6	60	70

**Table 2:** Summary of the material, electrical and optical properties of the films synthesized on the five different substrates: sapphire, SiO_2_/Si, AT-cut quartz, GaN/AlGaN/GaN/Si and muscovite.

Parameters	c-Plane Sapphire	SiO_2_/Si	AT-Cut Quartz	GaN/AlGaN/GaN/Si	Muscovite
RMS roughness of AFM image (nm)	8.19	7.37	10.3	9.75	12.2
2θ angles of prominent XRD peaks (FWHM)	38.36° (020) (0.14°)	69.28° (202) (0.06°)	38.5° (020) (0.20°)	38.52° (020) (0.16°)	36.8°
2θ angles of common XRD peaks (FWHM)	38.36° (020) (0.14°)	38.42° (202) (0.16°)	38.5° (020) (0.20°)	38.52° (020) (0.16°)	36.8°
Electrical Transition temperature (forward)	69 °C	76 °C	70 °C	67 °C	61 °C
Optical Transition temperature at λ = 1550 nm	64 °C	69 °C	59 °C	64 °C	79 °C
Resistance transition ratio	938	926	958	477	417
Change of Transmitted laser power or color at λ = 1550 nm (%)	80.84	82.8	81.86	85.33	80

## Appendix A

**Table A1:** Summary of the transmitted optical power change for VO_2_ films on sapphire and quartz substrates in the near infrared wavelength range.

Substrate	Reference	Synthesis Method	Wavelength (nm)	Film Thickness (nm)	Transmitted Power Change (%)	Crystal Type
Sapphire	Radue et al. [34]	Reactive bias target ion beam deposition	785	80	44.4	Polycrystalline
	**This paper**	**Direct oxidation**	**1550**	**140**	**80.84**	**Polycrystalline**
	Ma et al. [35]	Reactive magnetron sputtering	2000	20	33	Dual orientation
				50	50	
	Bian et al. [36]	Pulsed laser deposition	980	200	48	Monocrystalline
			1064	200	44	
	Zhang et al. [37]	DC magnetron sputtering	1550	140–185	45	Monocrystalline
Quartz	Liu et al. [31]	Reactive Pulsed laser ablation	1250	-	32	Amorphous
	Dejene et al. [8]	Reactive KrF laser ablation	1550	500	40.9	Polycrystalline
			1800	500	48.8	
	Zhang et al. [32]	DC magnetron sputtering	1550	140–185	41	-
	Radue et al. [34]	Reactive Biased Target Ion Beam Deposition	785	80	61.1	Polycrystalline
	Zhang et al. [29]	RF plasma assisted O-MBE	1550	60	40	Polycrystalline
	Zhao et al. [38]	Solution based route	1550		25	Polycrystalline
	Kang et al. [39]	Pulsed laser deposition	1550	60	20	Polycrystalline
	Bae son et al. [40]	IPL sintering	1550	145	30	Polycrystalline
	Houska et al. [41]	HiPMS	1550	80	27	Polycrystalline
	Long et al. [42]	Reactive magnetron sputtering	1550	80	35	Polycrystalline
	**This paper**	**Direct oxidation**	**1550**	**140**	**81.86**	**Polycrystalline**
SiO_2_/Si	**This paper**	**Direct oxidation**	**1550**	**140**	**82.8**	**Polycrystalline**
	Zhang et al. [37]	DC magnetron sputtering	1550	140–185	35	-
	Yu et al. [43]	RF magnetron sputtering	1550	80	37	Polycrystalline
	Kang et al. [44]	Solution processed synthesis	1550	150	35	Polycrystalline
	Luo et al. [45]	Reactive sputtering	2500	400	68	Polycrystalline
	Zhao et al. [46]	Solution processed synthesis	1550	100	35	Polycrystalline
GaN/AlGaN/GaN/Si	**This paper**	**Direct oxidation**	**1550**	**140**	**85.33**	**Polycrystalline**
	Zhang et al. [47]	Molecular beam epitaxy	2000	60	30	Polycrystalline
	Woo Yang [48]	RF magnetron sputtering	2400	100	70	Polycrystalline
Muscovite	**This paper**	**Direct oxidation**	**1550**	**140**	**80**	**Polycrystalline**
	Chen et al. [11]	Pulsed laser deposition	1550	100	25	Polycrystalline
